# The Effect of Electroacupuncture on the Extracellular Matrix Synthesis and Degradation in a Rabbit Model of Disc Degeneration

**DOI:** 10.1155/2014/731395

**Published:** 2014-05-27

**Authors:** Guo-fu Huang, Jing Zou, Jing Shi, Dong-you Zhang, Hong-fen Pen, Qi Zhang, Yu Gao, Bo-yi Wang

**Affiliations:** ^1^Department of Neurobiology, School of Basic Medicine, Tongji Medical College of Huazhong University of Science and Technology, 13 Hangkong Road, Wuhan 430030, China; ^2^Department of Acupuncture & Moxibustion, Wuhan Hospital of Integrated Chinese & Western Medicine, Tongji Medical College of Huazhong University of Science & Technology, 215 Zhongshan Road, Wuhan 430022, China

## Abstract

The present study was aimed at determining if the electroacupuncture (EA) is able to protect degenerated disc in vivo. New Zealand white rabbits (*n* = 40) were used for the study. The rabbits were randomly assigned to four groups. EA intervention was applied to one of the four groups. Magnetic resonance imaging and Pfirrmann's classification were obtained for each group to evaluate EA treatment on the intervertebral disc degeneration. Discs were analyzed using immunofluorescence for the labeling of collagens 1 and 2, bone morphogenetic protein-2 (BMP-2), matrix metalloproteinase-13 (MMP-13), and tissue inhibitor of matrix metalloproteinase-1 (TIMP-1). For protein expression analysis, western blot was used for biglycan and decorin. Outcomes indicated that EA intervention decreased the grades compared with the compressed disc. Immunofluorescence analysis showed a significant increase of collagens 1 and 2, TIMP-1, and BMP-2 positive cells, in contrast to MMP-13 after EA treatment for 28 days. The protein expression showed a sign of regeneration that decorin and biglycan were upregulated. It was concluded that EA contributed to the extracellular matrix (ECM) anabolic processes and increased the ECM components. MMPs and their inhibitors involved in the mechanism of EA intervention on ECM decreased disc. It kept a dynamic balance between ECM synthesis and degradation.

## 1. Introduction

Low-back pain is a global health problem due to its high prevalence and high socioeconomic burden. It affects 70 to 85% of the population during a lifetime and 15 to 45% in a year [1]. The main cause of low back pain is disc degeneration, of which the etiology is complex and multifactorial, involving age, genetics, and biomechanical and environmental factors such as immobilization, trauma, tobacco use, diabetes, vascular disease, and infection [2–5]. Although low-back pain constitutes a major public health issue, little is known about its precise mechanisms [6]. Nonsurgical treatment modalities currently available for symptomatic disc degeneration include lifestyle modifications, rehabilitation programs, and pain medications. Among the multiple patterns of treatments, acupuncture may have a favourable effect on self-reported pain and functional limitations induced by disc degeneration [7]. Since it originated from China, it has now become worldwide in its practice [8, 9]. Increasing statistics showed that a broader population has granted it acceptance. It was reported [10] that electroacupuncture (EA) inhibits AF cell apoptosis via the mitochondria-dependent pathway and upregulates Crk and ERK2 expression. Neuropeptide, a pain controller, produced by electrical acupuncture stimulation of different frequency [11]. The CB2 receptors also contribute to the analgesic effect of EA in a rat model of inflammatory pain [12, 13]. Although the analgesic effect of acupuncture is well documented, the biological basis is still not fully understood.

Lumbar intervertebral disc is a highly specialized structure composed of a complex system of various connective tissues. The abundant fibrils of intervertebral discs are collagen type 1 and 2. As an important matrix component, the predominant proteoglycan, including decorin, biglycan, ibromodulin, and perlecan were found in the nucleus pulposus [14]. Proteoglycans provide the swelling pressure required to confer a high swelling propensity for load support and collagens resist to the volume increase involved in swelling [15–17]. One of its main functions of intervertebral disc is dampening compressive loads. Depending on the duration and extent of the loading, it leads to significant degeneration [16, 18], thus breaking the balance between extracellular matrix (ECM) synthesis and degradation and resulting in a gradual loss of disc extracellular matrix and, eventually, structural failure [19].

The purpose of the current study was to determine and evaluate the effect of EA on the recovery of disc degeneration. Firstly a custom-made dynamic disc compression device was used to induce a disc degeneration model of rabbits, and then the rabbits received EA administration. For this purpose, Pfirrmann's MRI grade scores were obtained for disc degeneration, and a quantitative molecular and histology analysis was used for (1) extracellular matrix components, including COL-1 and COL-2, biglycan, and decorin; (2) extracellular matrix regulatory factors, catabolic factors, and their inhibitors, including MMP-13, TIMP-1, and BMP-2.

## 2. Materials and Methods

### 2.1. Animals

All animal procedures were performed under the approval and guidance of the Animal Care and Use Committee at Wuhan Hospital of Integrated Chinese & Western Medicine, affiliated to Tongji Medical College of Huazhong University of Science & Technology. A total of 40 New Zealand skeletally mature white rabbits (3.5–4 kg) were used for the study. The rabbits were randomly assigned to four groups, and ten for each group were given different interventions at 28-day and 56-day time point [20, 21]. Both the compression group (*n* = 10) and the EA group (*n* = 10) were first loaded for 28 days using a custom-made external compression device to stimulate disc degeneration. After 28-day loading time, in the compression group, five were killed and the tissue was harvested, with the other five using the same device for another 28 days. In EA group, tissue was harvested for five rabbits, and the other five received EA administration for 28 days after removal of the external device. In sham compression group (*n* = 10), the rabbits received surgical preferment, but the lumbar body was only punctured without previous loading for 28 days (*n* = 5) or 56 days (*n* = 5). Ten rabbits, which served as controls, were normally fed without surgical preferment for 28 days (*n* = 5) or 56 days (*n* = 5).

### 2.2. Surgical Procedure

**Figure 1:**
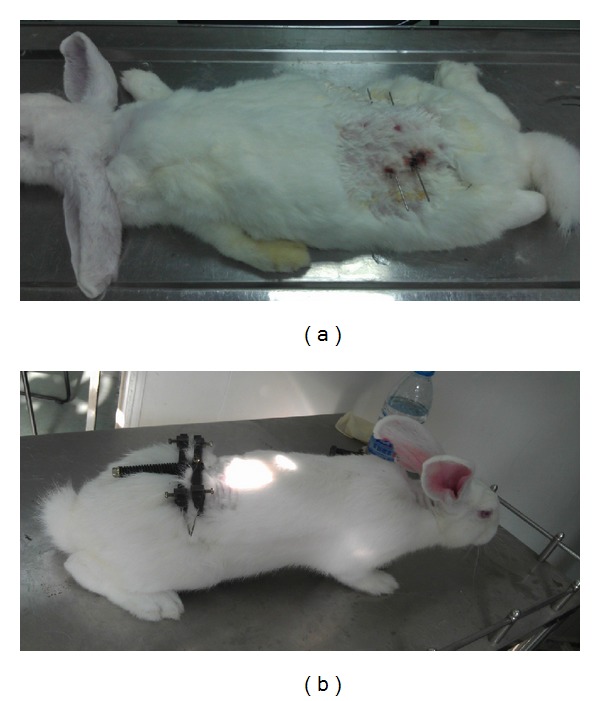
(a) Two K-wires (1.5 mm diameter) inserted into the vertebral body. (b) External dynamic compression device attached to the rabbit lumbar spine.

Rabbits were anesthetized with 10% chloral hydrate administered via the marginal ear vein. Through a dorsal approach to the lumbar spine, the custom-made external device was attached to two K-wires (1.5 mm diameter) inserted into the vertebral bodies L4 and L5 parallel to the adjacent study disc by the use of a variable-speed electric drill [22] (Figure 1(a)). After the wound was closed, in 20 animals, axial compression to the disc was created by a spring within the device to produce a disc compression force of 200 N to induce disc degeneration (Figure 1(b)). The sham compression group was performed the same way, but the external compression device was placed in situ without application of compressive force.

### 2.3. Magnetic Resonance Imaging

Magnetic resonance imaging (MRI) was obtained for each group at days 28 and 56. Imaging was performed at 30 minutes after removal of the external fixateur to establish a new hydration equilibrium of the disc. A custom-made positioning device consisting of foamed material was used to achieve a standardized supine position of the animal. MRI was performed with a 3.0 T imager (GE, American) with a synergy spine coil receiver. T2-weighted sections in the sagittal plane were obtained in the following settings: fast spin echo sequence and time to repetition (TR) of 2200 milliseconds; time to echo (TE) of 70.7 milliseconds; matrix 336 (h) ∗ 512 (v); field of view at 120 mm; 8 excitations; section thickness of 2 mm; gap of 0.2 mm (T1: TR 375; TE 15; matrix 304 (h) ∗ 512 (v); 18 excitations). Pfirrmann's classification [23] was used for disc degeneration grading from grade 1 to 5 (1 = normal, 2 = mild degeneration, 3 = moderate degeneration, 4 = severe degeneration, and 5 = advanced degeneration).

### 2.4. EA Treatment

In the EA treatment group, five of the rabbits received EA administration on the Ex-B2 (paravertebral point of L4 and L5 level on both sides) once every day, starting at the second day after the device was removed, and lasted for 28 days. Four acupuncture needles were inserted into 4 acupoints that correspond to Ex-B2 in the rabbits; EA (1 mA and 0.4 or 0.6 ms) was administered at 2 or 15 Hz for 30 minutes. Current was delivered with modified current-constant Han's Acupoint Nerve Stimulator (Beijing, China). Ex-B2 were chosen according to the traditional Chinese medicine meridian theory and the effective use in reducing pain. During EA treatment, each rabbit was placed under an inverted clear wooden box (approximately 40 × 25 × 40 cm) but was neither restrained nor given any anesthetic. The animals remained awake and still during EA treatment and showed no evident signs of distress.

### 2.5. Tissue Preparation

After 28 or 56 days of different intervention, the lumbar disc was harvested for examination of each group, including complete anulus fibrosus and nucleus pulposus. Using a vertical midline incision, the disc was divided into 2 symmetric parts. One part was immediately quick frozen in liquid nitrogen for protein expression analysis; the second part was used for immunofluorescence.

### 2.6. Immunofluorescence

Disc samples were fixed in formalin 4%, serially dehydrated in ethanol, and embedded in paraffin. The paraffin blocks were sectioned transversely at a 5 m thickness using a standardized manner to ensure that each slide was obtained from the same disc area. Tissue sections were washed with 5% Tween 20 in PBS for three times, incubated in 1 N HCL for 20 min and in 3% H_2_O_2_ in distilled water for 15 min, and blocked with 5% goat serum for 30 min at room temperature to block the unspecific staining, respectively. The sections were incubated with a mixture solution of primary antibodies, antihuman COL-1 (Biorbyt, UK), antihuman COL-2 (Biorbyt, UK); antihuman BMP-2 (Bioss, Beijing, China); antihuman MMP-13 (Boster, Wuhan, China); antihuman TIMP-1 (Bioss, Beijing, China) for 48 h at 4°C, washed three times in PBS, and incubated in the secondary antibody, goat antirabbit immunoglobulin G (red fluorescence, diluted 1 : 300; Boster, Wuhan), on a rocking bed (away from light) for 2 h under room temperature, respectively. For control staining, primary antibody was omitted. The tissue sections were mounted on glass slides, washed four more times with running water, dried under room temperature and away from light, and sealed with coverslips at last. The analysis was performed using a light microscope NIKON Eclipse 80i with an objective magnification of 200x and software Analysis Pro 3.1. Visualization was performed with avidin-biotin complex method. The fluorescence intensity was measured by Image-Pro Plus 6.0 (USA).

### 2.7. Western Blotting Analysis

Total protein was extracted from the tissue in RIPA lysis buffer (containing protease and phosphatase inhibitor mixtures) by using a tissue homogenizer, followed by clearing tissue debris by centrifugation at 13000 rpm at 4°C for 20 min. Fifty micrograms of protein were loaded per lane and separated by 10% SDS-PAGE gel electrophoresis and, then, transferred onto PVDF membranes. Blocking was carried out in 5% nonfat dry milk in Tris-buffered saline (TBS) containing 0.1% Tween 20 for 1 h at room temperature. The membranes were incubated with primary antibody rabbit antidecorin (diluted 1 : 200; Boster, Wuhan, China); anti-biglycan (diluted 1 : 200; Bioss, Beijing, China) over night at 4°C and with secondary antibody (1 : 40000 dilution of goat antirabbit Immunoglobulin G) conjugated to horseradish peroxidase (Boster, Wuhan, China) for 1 h at room temperature on the following day. Immunoblotting signal was detected by ECL (enhanced chemiluminescence) on chemiluminescent films following exposure to an X-ray. For densitometric analyses, the blots were scanned and quantified using BandScan software, and the result was expressed as the ratio of target gene immunoreactivity to GAPDH immunoreactivity.

### 2.8. Statistical Analysis

The data collected in the present study were expressed as mean ± standard deviation (mean ± SD) and analyzed by one-way repeated measures ANOVA to determine differences between two groups. *P* < 0.05 was considered statistically significant.

## 3. Results

### 3.1. The Effect of EA on MRI Grade Scores in Disc Degeneration

**Figure 2:**
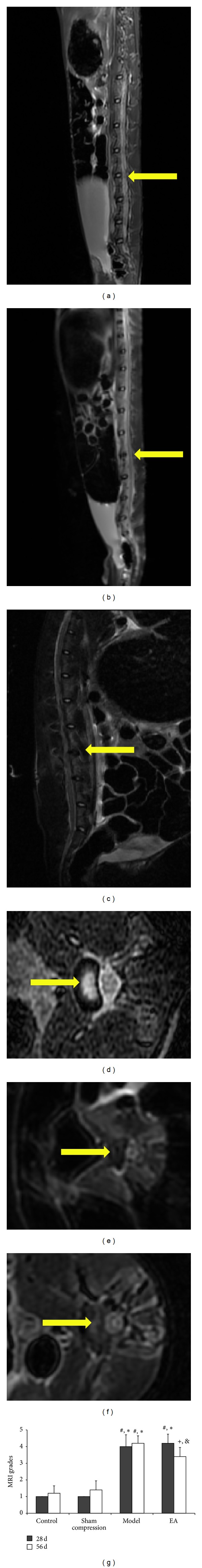
Representative T2-weighted sagittal MRI of the nucleus pulposus at the 56-day time point shows lower signal intensity in model group (b) and EA group (c) than the control group (a). (d), (e), and (f) are the corresponding MRI axial scan, respectively, to (a), (b), and (c). Different signal intensity in the disc is depicted with arrows. Change in disc degeneration of four groups (g). Pfirrmann's classification based on disc height and signal intensity from grade 1 to 5 was used to grade the disc degeneration of the rabbit discs. Data are expressed as the mean ± SD (ANOVA); *P < 0.05, compared with the control group; ^#^ *P* < 0.05, compared with sham compressed group; ^+^ *P* < 0.05, compared with the model group on 56 days and compared with 28 days in EA group.

The MRI assessment showed that the healthy and compressed discs are clearly differentiated on the T2-weighted image, and the signal intensity of the nucleus pulposus decreased progressively during the 28-day compression period, with the lowest signal intensity after compression for 56 days. Different images showed that the device was able to induce the IVD model. According to Pfirrmann's MRI grade scores, which indicate the degree of disc degeneration, grade IV degenerative changes were first detected at 28 days after compression, and grade IV or V was detected 56 days after loading. In contrast, the control and sham groups remained relatively constant during the 28- or 56-day period, with grade I on T2-weighted imaging. After EA intervention for 28 days, the degree of degenerated disc was characterized by grade III or IV, compared to the model group in 28 days and 56 days and EA group in 28 days (*P* < 0.05) (Figure 2).

### 3.2. The Effects of EA on ECM Components in Disc Degeneration

**Figure 3:**
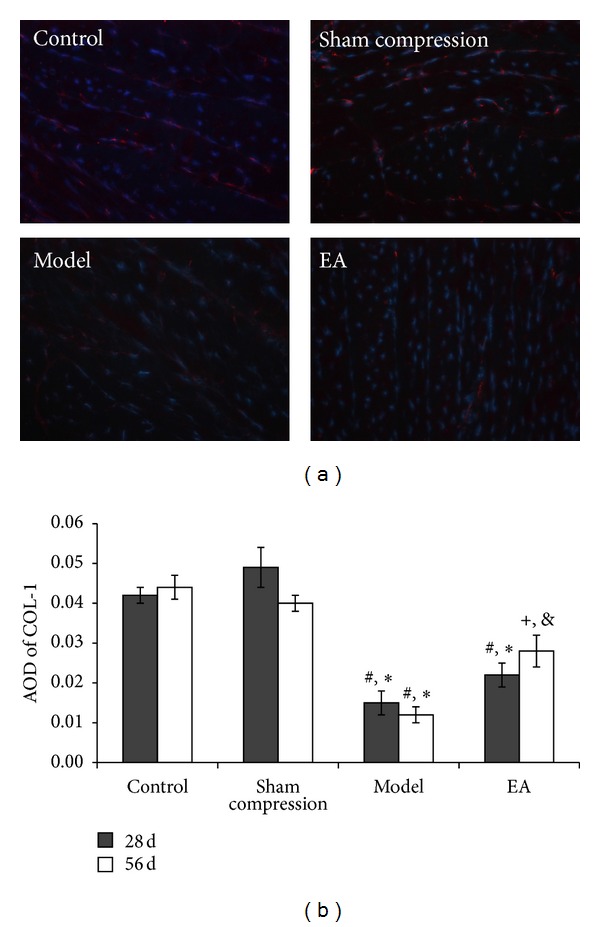
(a) Representative microscopic photos of immunofluorescence staining showing COL-1 immunoreaction (IR) positive products. Nucleus counter stained with DAPI showed blue and red for COL-1 positive products. (b) Average optical density (AOD) was measured and data are expressed as the mean ± SD (ANOVA); *P < 0.05, compared with the control group; ^#^ *P* < 0.05, compared with sham compressed group, ^+^ *P* < 0.05, compared with the model group on 56 days and compared with 28 days in EA group.

**Figure 4:**
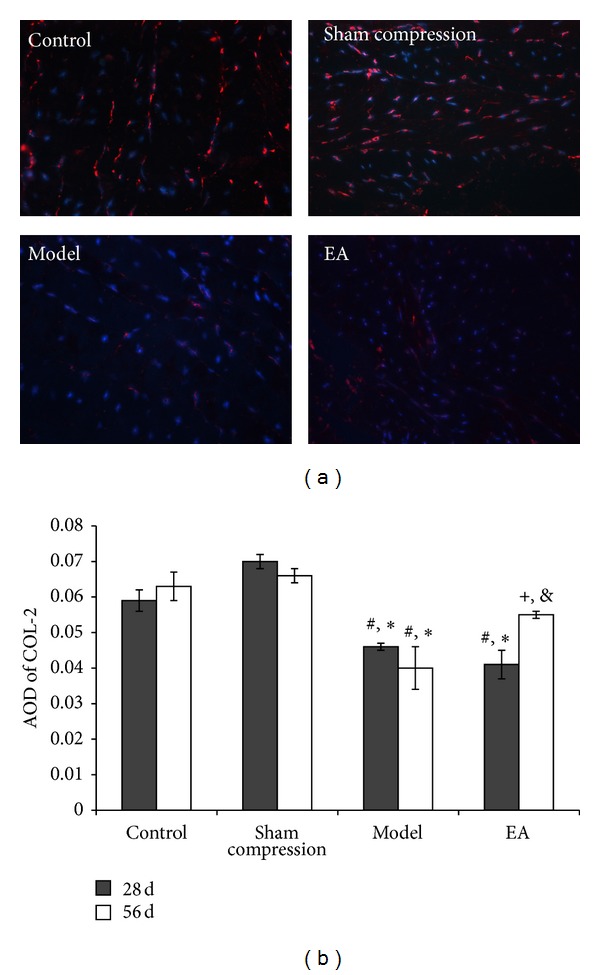
(a) Immunofluorescence staining showing COL-2 immunoreaction (IR) positive products. Nucleus counter stained with DAPI showed blue and red for COL-2 positive products. (b) AOD was measured and data are expressed as the mean ± SD (ANOVA); *P < 0.05, compared with the control group; ^#^ *P* < 0.05, compared with sham compressed group, ^+^ *P* < 0.05, compared with the model group on 56 days and compared with 28 days in EA group.

Immunofluorescence labeling was used to detect the immunoreactivity of COL-1 (Figure 3) and COL-2 (Figure 4). In comparison with the control group and the sham compression group, the immunoactivity levels of COL-1 and COL-2 in the compressed disc were found to be decreased significantly (*P* < 0.05). After EA intervention, the immunoreactions positive cells in the 56 days of EA group were found to be obviously higher than that in the model group and the 28 days in the EA group (*P* < 0.05).

**Figure 5:**
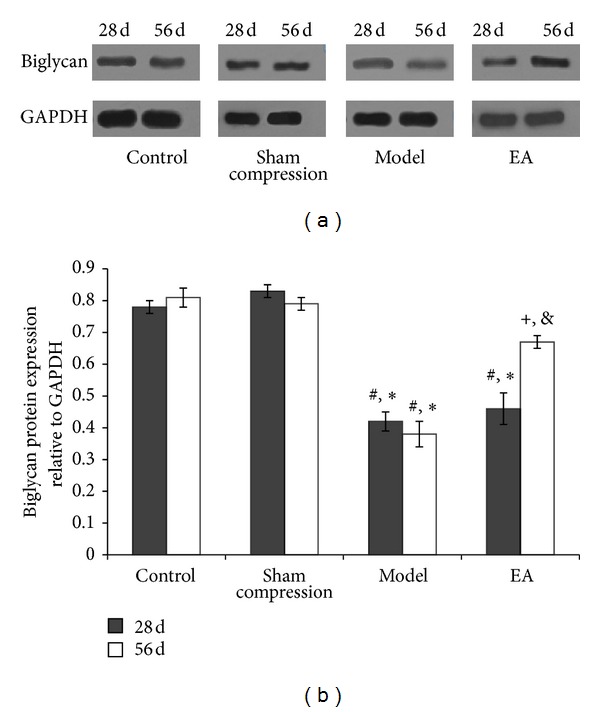
(a) Representative western blot analyses showing the biglycan protein levels. GAPDH was analyzed as house-keeping gene. (b) Protein expression of four groups. *P < 0.01, compared with the normal control; ^#^ *P* < 0.05, compared with the sham compressed group, ^+^ *P* < 0.05, compared with the model group on 56 days and compared with 28 days in EA group.

**Figure 6:**
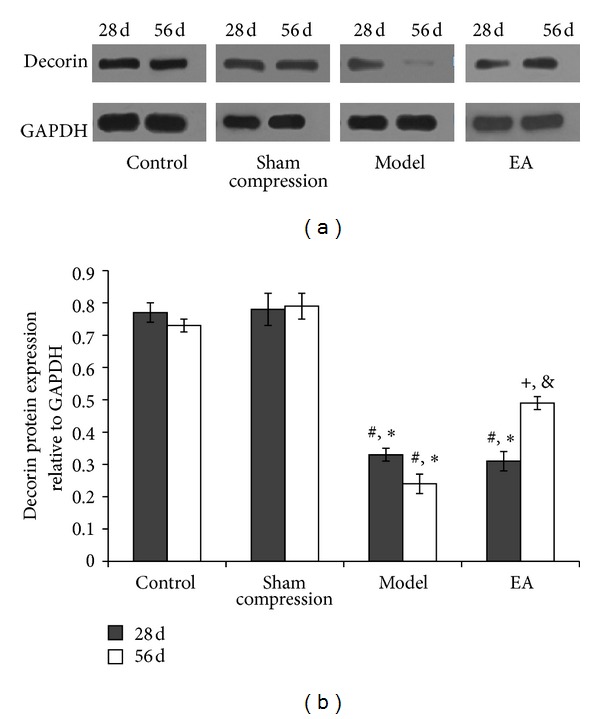
(a) Representative western blot analyses showing the decorin protein levels. GAPDH was analyzed as house-keeping gene. (b) The protein expression of four groups. *P < 0.05, compared with the normal control; ^#^ *P* < 0.05, compared with the sham compressed group, ^+^ *P* < 0.05, compared with the model group on 56 days and compared with 28 days in EA group.

Western blot analysis demonstrated that no significant changes were found between control and sham compression groups at any time point. Compared with the two groups, the relative expression levels of biglycan (Figure 5) (*P* < 0.01) and decorin (Figure 6) (*P* < 0.05) protein in disc were apparently decreased in the model group. Following EA intervention, the expression in the EA group was considerably higher than those in the model group and the 28 days in the EA group. (*P* < 0.05). A trend to stimulated expression was found in matrix components.

### 3.3. The Effect of EA on ECM Regulatory Factor in Disc Degeneration

**Figure 7:**
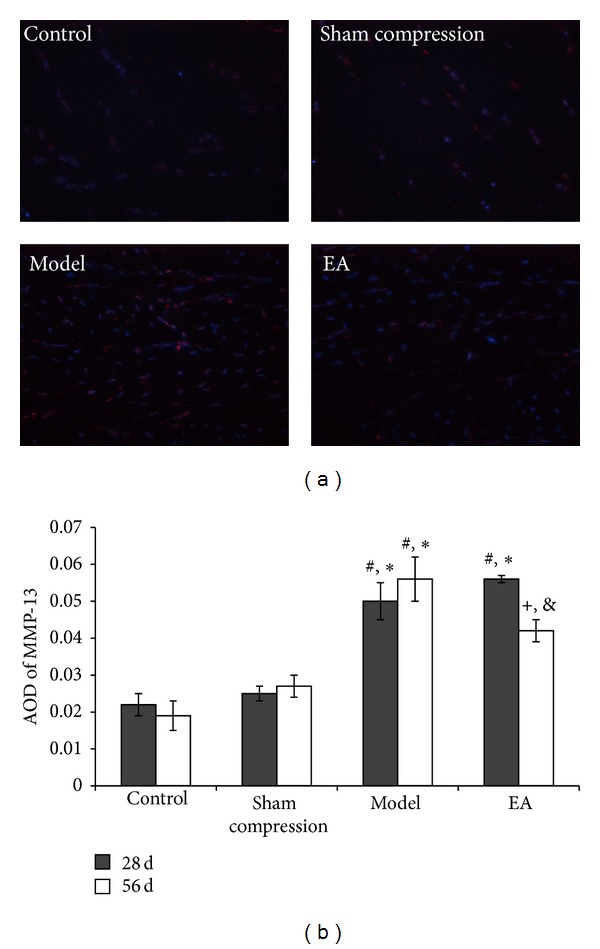
(a) Nucleus counter stained with DAPI showed blue and red for MMP-13 positive products. (b) AOD was measured and data are expressed as the mean ± SD (ANOVA); *P < 0.01, compared with the control group; ^#^ *P* < 0.05, compared with the sham compressed group,^+^ *P* < 0.05, compared with the model group on 56 days and compared with 28 days in EA group.

MMPs are a family of inducible, zinc-dependent, secreted, or cell surface based endopeptidases that are centrally involved in the turnover of extracellular matrix (ECM) components. MMP-13, also known as collagenase-3, is the principal interstitial collagenase in this species and has a high specificity for degrading insoluble fibrillar collagens, especially types II and I collagens [24, 25]. In contrast, TIMP-1 is an endogenous inhibitor of bone matrix degradation that binds tightly to active MMP-13, thereby downregulating MMP-13 activity. BMP-2, one of the growth factors, has been found to be capable of enhancing cell proliferation and ECM synthesis in vitro and in vivo. We detected MMP-13 by the immunofluorescence method and found that MMP-13 immunoreactivity in the compressed disc was increased compared with the control group, of which the positive cells were not even detectable (*P* < 0.05). Following EA intervention, the immunoreactivity level was downregulated (*P* < 0.05) (Figure 7).

**Figure 8:**
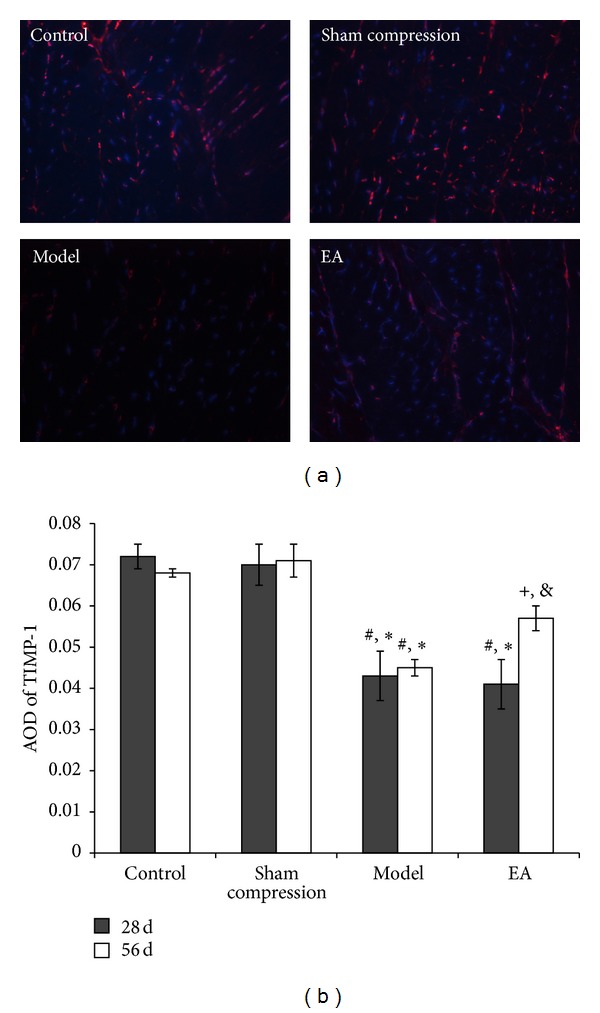
(a) Nucleus counter stained with DAPI showed blue and red for TIMP-1 positive products. (b) AOD was measured and data are expressed as the mean ± SD (ANOVA); *P < 0.05, compared with the control group; ^#^ *P* < 0.05, compared with the sham compressed group, ^+^ *P* < 0.05, compared with the model group on 56 days and compared with 28 days in EA group.

**Figure 9:**
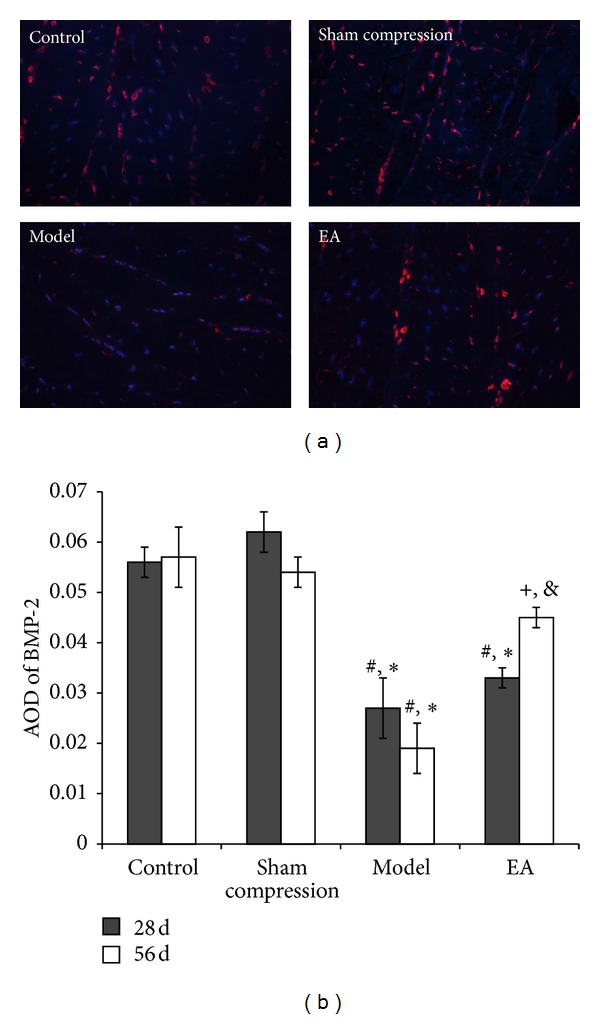
(a) Nucleus counter stained with DAPI showed blue and red for BMP-2 positive products. (b) AOD was measured and data are expressed as the mean ± SD (ANOVA); *P < 0.01, compared with the control group; ^#^ *P* < 0.01, compared with sham compressed group,^+^ *P* < 0.05, compared with the model group on 56 days and compared with 28 days in EA group.

TIMP-1, inhibitor of ECM catabolic factors, and the growth factor BMP-2 were detected by the same method. Compared with the control group and the sham group, the immunoreactivity level of TIMP-1 was lower in the compressed disc (*P* < 0.05), which was in contrast to the MMP-13 (Figure 8). After EA intervention, immunoreactivity level was upregulated in comparison with the model group (*P* < 0.05). The result of BMP-2 was similar to that observed in TIMP-1 (Figure 9).

## 4. Discussion

At present, the methods available to delay degeneration of intervertebral discs include direct injection of cytokines, cell transplantation into intervertebral discs, or tissue engineering. Increasing attention has been paid to the regeneration of functional tissue based on the restoration of the ECM integrity by cell therapy [26, 27]. However, both current nonsurgical treatment modalities and surgical options for severe symptomatic intervertebral disc degeneration have limited and inconsistent clinical results [28]. EA treatments are effective approaches, which offer the potential to halt, retard, or even reverse disc degeneration and restore physiologic disc function. Our study provides new information about the mechanisms underlying the protected effect of EA on disc degeneration. The present study indicated that EA had anabolic and anticatabolic effects on the regulation of extracellular matrix in IVD degeneration model as assessed by MRI, western blot, and immunofluorescence analyses.

First, the current results from the imaging studies support the opinion that EA intervention resulted in a number of slowly progressive and reproducible MRI changes over 28 days. MRI technique allows the definition of IVD based on the tissue hydration shown by the intensity of the T2ws in the NP and various classification systems [29, 30]. It was the gold standard for the clinical investigation of IVD integrity in humans and animal study. The pictures of rabbit lumbar spines in the research showed a significant decrease of nucleus pulposus hydration after 28 days of compression, in contrast to sham compression or controlled discs. Thus indicating a loading-dependent loss and the appearance of a dark transverse band. This data is quite similar to that observed in humans during the course of IVD degeneration. On the other hand, Pfirrmann's classification system was used to assess the effect of EA on the degree of degeneration. It was found that the degeneration grade on MRI was significantly decreased after EA treatment compared with the compressed disc. These findings demonstrate the effectiveness of the EA intervention in a disc degeneration animal model.

Second, we have shown that EA increases extracellular matrix protein expression and the immunoreactivity level, of which biglycan, decorin, COL-1, and COL-2 were significantly stimulated when compared with compressed discs. Histology and protein analysis are consistent with the abovementioned MRI findings. This sequence helps understand the fact that the intervertebral disc degeneration is characterised by ECM decrease, resulting from an imbalance between the anabolic and catabolic processes [31]. It was known that the intervertebral disc consists of COL-1 in the outer anulus fibrosus and the widespread COL-2. Small proteoglycans are represented by decorin in the anulus fibrosus and biglycan in the nucleus pulposus [32]. Those are the important composition of ECM. Its major function is water binding capacity affected by negatively charged glycosaminoglycans [33]. The amount of ECM is dependent on the balance between its production and digestion, and this is a sort of state of dynamic equilibrium [34, 35]. Studies [36] have reported that distraction resulted in stimulated ECM gene expression and increased numbers of protein-expressing cells, showing evidence of regenerative potential. In the current study we achieved the similar result and found that EA method plays the same role as distraction in disc repair. The immunofluorescence findings showed confirmatory characteristics of disc renewal and improved lamellar architecture after EA treatment for 28 days, and the number of COL-1 and COL 2 positive cells was significantly increased. And also the protein expression of biglycan and decorin in the EA group was considerably higher than those in the model group and the 28 days in the EA group. These results suggest that the nutrient supply in the disc was increased by EA treatment, particularly for proteoglycan content.

Third, we studied the role of EA played in the ECM regulatory factors, including MMP-13, TIMP-1, and BMP-2, in disc degeneration. It is likely that ECM regulatory factors involved in the mechanism of EA intervention on disc ECM decrease.

Matrix metalloproteinases (MMPs) and inhibitors of MMPs, a kind of enzymes which mediate the catabolic process, have been reported to play a major role in the disc degeneration process [37–40], which are stimulated at an early stage of disease and initiate matrix degradation. MMP-13, known as one of the markers for degradation, is probably the most trustworthy among the various MMPs proteins modulated in osteoarthritic chondrocytes. The increase in MMP13 could therefore be a major contributor to IVD degeneration as it has been extensively reported in cartilage degradation during OA [41, 42]. In ECM metabolism, much evidence for TIMP functions has been accumulated [43, 44]. TIMPs inhibit MMPs by 1 : 1 interaction with zinc-binding site [44]. TIMP-1 is a known endogenous inhibitor of MMP-1 and MMP-3 [45]. BMP-2 is a potent osteoinductive agent [46] and plays a major role as a growth factor during early chondrogenesis [47]. Previous study [36] supports that BMP-2 was involved in disc metabolism and may reflect anabolic behavior in the disc reorganization process. In vitro delivery of the anticatabolic genes TIMP-1 and BMP-2 cause the increase of proteoglycans in cultured degenerated human disc cells. In our study, a positive MMP-13 result was found in compressed disc, whereas, in control or sham compression discs, the immunoreaction positive cells were not even detectable. Following EA intervention for 28 days, the immunoreactivity level decreased compared with the discs loaded for 28 or 56 days. However, the expressions of TIMP-1 and BMP-2 are totally different from MMP-13. Although a causative role has not been proved, our results support the hypothesis that ECM regulatory factors participate in the reorganization process of disc disease treated with the EA method.

In summary, the author performed this in vivo study to determine the effect of EA treatment in a degenerated disc rabbit model. The results in images confirmed that EA reduced Pfirrmann's MRI grade scores. EA increased BMP-2 and TIMP-1 and decreased MMP-13 in the immunoreactivity or protein leve; subsequently it enhances the synthesis of matrix proteoglycan, diminishes the degradation of COL-1 and-2 in disc tissues, and, eventually, showed the anabolic effect on degenerated discs. These results indicate that EA therapy has significant potential for treatment of degenerative disc disease. However, further research is needed on the specific signaling pathways mediated by EA method.

## Abbreviations

EA: Electroacupuncture
MRI: Magnetic resonance imaging
BMP: Bone morphogenetic protein
MMPs: Matrix metalloproteinases;
TIMPs: Tissue inhibitor of matrix metalloproteinases
COL: Collagen
ECM: Extracellular matrix
GAPDH: Glyceraldehyde 3-phosphate dehydrogenase
PBS: Phosphate buffered saline
FITC: Fluorescein isothiocyanate
AOD: Average optical density.

## References

[1] Andersson GBJ (1999). Epidemiological features of chronic low-back pain. *The Lancet*.

[2] Battie MC, Videman T, Gibbons LE, Fisher LD, Manninen H, Gill K (1995). Determinants of lumbar disc degeneration: a study relating lifetime exposures and magnetic resonance imaging findings in identical twins. *Spine*.

[3] Lotz JC, Colliou OK, Chin JR, Duncan NA, Liebenberg E (1998). Compression-induced degeneration of the intervertebral disc: an in vivo mouse model and finite-element study. *Spine*.

[4] Lotz JC, Staples A, Walsh A, Hsieh AH Mechanobiology in intervertebral disc degeneration and regeneration.

[5] Pye SR, Reid DM, Adams JE, Silman AJ, O’Neill TW (2007). Influence of weight, body mass index and lifestyle factors on radiographic features of lumbar disc degeneration. *Annals of the Rheumatic Diseases*.

[6] Zhang Y, An HS, Tannoury C, Thonar EJ-MA, Freedman MK, Anderson DG (2008). Biological treatment for degenerative disc disease: implications for the field of physical medicine and rehabilitation. *American Journal of Physical Medicine and Rehabilitation*.

[7] Lam M, Curry P, Galvin R (2013). The effectiveness of acupuncture for non-specific chronic low back pain: a systematic review and meta-analysis. *Spine*.

[8] (1998). NIH Consensus Conference. Acupuncture. *The Journal of the American Medical Association*.

[9] Li A, Kaptchuk TJ (2011). The case of acupuncture for chronic low back pain: when efficacy and comparative effectiveness conflict. *Spine*.

[10] Goldman N, Chen M, Fujita T (2010). Adenosine A1 receptors mediate local anti-nociceptive effects of acupuncture. *Nature Neuroscience*.

[11] Han J-S (2003). Acupuncture: neuropeptide release produced by electrical stimulation of different frequencies. *Trends in Neurosciences*.

[12] Chen L, Zhang J, Li F (2009). Endogenous anandamide and cannabinoid receptor-2 contribute to electroacupuncture analgesia in rats. *Journal of Pain*.

[13] Neufeld JH (1992). Induced narrowing and back adaptation of lumbar intervertebral discs in biomechanically stressed rats. *Spine*.

[14] Roughley PJ (2004). Biology of intervertebral disc aging and degeneration: involvement of the extracellular matrix. *Spine*.

[15] Oegema TR (1993). Biochemistry of the intervertebral disc. *Clinics in Sports Medicine*.

[16] Oegema TR (2002). The role of disc cell heterogeneity in determining disc biochemistry: a speculation. *Biochemical Society Transactions*.

[17] Scott JE, Bosworth TR, Cribb AM, Taylor JR (1994). The chemical morphology of age-related changes in human intervertebral disc glycosaminoglycans from cervical, thoracic and lumbar nucleus pulposus and annulus fibrosus. *Journal of Anatomy*.

[18] Yamada K (1962). The dynamics of experimental posture. Experimental study of intervertebral disk herniation in bipedal animals. *Clinical Orthopaedics*.

[19] Buckwalter JAMVC, Boden SD, Eyre DR, Weidenbaum M (2000). Intervertebral disk structure, composition, and mechanical function. *Orthopaedic Basic Sciences: Biology and Biomechanics of the Musculoskeletal System*.

[20] Cassidy JD, Yong-Hing K, Kirkaldy-Willis WH, Wilkinson AA (1988). A study of the effects of bipedism and upright posture on the lumbosacral spine and paravertebral muscles of the Wistar rat. *Spine*.

[21] Gloobe H, Nathan H (1973). Osteophyte formation in experimental bipedal rats. *Journal of Comparative Pathology*.

[22] Kroeber MW, Unglaub F, Wang H (2002). New in vivo animal model to create intervertebral disc degeneration and to investigate the effects of therapeutic strategies to stimulate disc regeneration. *Spine*.

[23] Pfirrmann CWA, Metzdorf A, Zanetti M, Hodler J, Boos N (2001). Magnetic resonance classification of lumbar intervertebral disc degeneration. *Spine*.

[24] Vincenti MP, Brinckerhoff CE (2002). Transcriptional regulation of collagenase (MMP-1, MMP-13) genes in arthritis: integration of complex signaling pathways for the recruitment of gene-specific transcription factors. *Arthritis Research*.

[25] Uchinami H, Seki E, Brenner DA, D’Armiento J (2006). Loss of MMP 13 attenuates murine hepatic injury and fibrosis during cholestasis. *Hepatology*.

[26] Clouet J, Vinatier C, Merceron C (2009). The intervertebral disc: from pathophysiology to tissue engineering. *Joint Bone Spine*.

[27] Kalson NS, Richardson S, Hoyland JA (2008). Strategies for regeneration of the intervertebral disc. *Regenerative Medicine*.

[28] Lee CK (1988). Accelerated degeneration of the segment adjacent to a lumbar fusion. *Spine*.

[29] Sobajima S, Kompel JF, Kim JS (2005). A slowly progressive and reproducible animal model of intervertebral disc degeneration characterized by MRI, X-ray, and histology. *Spine*.

[30] Thalgott JS, Albert TJ, Vaccaro AR (2004). A new classification system for degenerative disc disease of the lumbar spine based on magnetic resonance imaging, provocative discography, plain radiographs and anatomic considerations. *Spine Journal*.

[31] Weiler C, Nerlich A, Zipperer J, Bachmeier B, Boos N (2002). 2002 SSE award competition in basic science: expression of major matrix metalloproteinases is associated with intervertebral disc degradation and resorption. *European Spine Journal*.

[32] Cs-Szabo G, Ragasa-San Juan D, Turumella V, Masuda K, Thonar EJ-MA, An HS (2002). Changes in mrna and protein levels of proteoglycans of the anulus fibrosus and nucleus pulposus during intervertebral disc degeneration. *Spine*.

[33] Urban JPG, Roberts S (2003). Degeneration of the intervertebral disc. *Arthritis Research and Therapy*.

[34] Stokes IAF, Iatridis JC (2004). Mechanical conditions that accelerate intervertebral disc degeneration: overload versus immobilization. *Spine*.

[35] Kroeber M, Unglaub F, Guehring T (2005). Effects of controlled dynamic disc distraction on degenerated intervertebral discs: an in vivo study on the rabbit lumbar spine model. *Spine*.

[36] Guehring T, Omlor GW, Lorenz H (2005). Stimulation of gene expression and loss of anular architecture caused by experimental disc degeneration—an in vivo animal study. *Spine*.

[37] Goupille P, Jayson MIV, Valat J-P, Freemont AJ (1998). Matrix metalloproteinases: the clue to intervertebral disc degeneration?. *Spine*.

[38] Doita M, Kanatani T, Ozaki T, Matsui N, Kurosaka M, Yoshiya S (2001). Influence of macrophage infiltration of herniated disc tissue on the production of matrix metalloproteinases leading to disc resorption. *Spine*.

[39] Shen B, Melrose J, Ghosh P, Taylor TKF (2003). Induction of matrix metalloproteinase-2 and -3 activity in ovine nucleus pulposus cells grown in three-dimensional agarose gel culture by interleukin-1*β*: a potential pathway of disc degeneration. *European Spine Journal*.

[40] Razaq S, Wilkins RJ, Urban JPG (2003). The effect of extracellular pH on matrix turnover by cells of the bovine nucleus pulposus. *European Spine Journal*.

[41] Goldring MB, Otero M, Tsuchimochi K, Ijiri K, Li Y (2008). Defining the roles of inflammatory and anabolic cytokines in cartilage metabolism. *Annals of the Rheumatic Diseases*.

[42] le Maitre CL, Freemont AJ, Hoyland JA (2007). Accelerated cellular senescence in degenerate intervertebral discs: a possible role in the pathogenesis of intervertebral disc degeneration. *Arthritis Research and Therapy*.

[43] Nagase H, Kashiwagi M (2003). Aggrecanases and cartilage matrix degradation. *Arthritis Research and Therapy*.

[44] Visse R, Nagase H (2003). Matrix metalloproteinases and tissue inhibitors of metalloproteinases: structure, function, and biochemistry. *Circulation Research*.

[45] Gomis-Ruth FX, Maskos K, Betz M (1997). Mechanism of inhibition of the human matrix metalloproteinase stromelysin-1 by TIMP-1. *Nature*.

[46] Boden SD, Kang J, Sandhu H, Heller JG (2002). Use of recombinant human bone morphogenetic protein-2 to achieve posterolateral lumbar spine fusion in humans: a prospective, randomized clinical pilot trial 2002 volvo award in clinical studies. *Spine*.

[47] Kim D-J, Moon S-H, Kim H (2003). Bone morphogenetic protein-2 facilitates expression of chondrogenic, not osteogenic, phenotype of human intervertebral disc cells. *Spine*.

